# Reinduction of Hormone Sensitivity to Goserelin following Chemotherapy with Vinorelbine in Castration-Resistant Prostate Cancer

**DOI:** 10.1100/tsw.2010.163

**Published:** 2010-09-14

**Authors:** Tal Grenader, Anthony Goldberg

**Affiliations:** Department of Oncology, Sha'are Zedek Medical Center, Jerusalem, Israel

**Keywords:** hormone-resistant prostate cancer, luteinizing hormone releasing hormone (LHRH) agonists, chemotherapy, vinorelbine

## Abstract

Primary androgen ablation leads to symptomatic improvement and a reduction in prostate-specific antigen (PSA) serum levels in patients with advanced prostate cancer, but all patients eventually become refractory to hormone therapy with progression of the disease and a life expectancy of about a year. We describe a patient who developed castration resistance, was treated with vinorelbine, and continues to be progression free on therapy with luteinizing hormone releasing hormone agonists alone, more than 2.5 years following cessation of treatment with vinorelbine.

## INTRODUCTION

Despite the more frequent diagnosis and treatment of prostate cancer at an early stage, the majority of men in whom the disease recurs does not have a potentially curative treatment option. The generally accepted treatment for advanced prostate cancer involves either surgical castration or chemical castration. The latter uses luteinizing hormone releasing hormone (LHRH) agonists or a combination of an LHRH agonist together with an antiandrogen. In approximately 80% of men, primary androgen ablation leads to symptomatic improvement and a reduction in prostate-specific antigen (PSA) serum levels, but all patients eventually become refractory to hormone therapy with progression of the disease and a life expectancy of about a year. Once the patient has become castration resistant, chemotherapy remains a viable option and the current treatment of choice in these circumstances is docetaxel based[1,2]. It is important, however, to have a range of suitable agents available as the elderly (who comprise the majority of prostate cancer patients) generally have a poor tolerance to these drugs. Vinorelbine may be useful in this context as it has a favorable side effects profile[3]. There is emerging evidence to suggest that in some cases, reinduction of hormone sensitivity may develop following chemotherapy[4,5,6]. We describe a patient who developed hormone resistance, was treated with vinorelbine, and continues to be progression free on hormone therapy alone, more than 2 years following cessation of treatment with vinorelbine.

## CASE REPORT

A 73-year-old male patient was admitted to the ward in March 2005 complaining of lower back pain. He had a previous history of coronary artery disease and myocardial infarction, following which he had undergone a coronary artery bypass graft in 2000.

Laboratory investigations on admission revealed a PSA of 335 ng/mL, and a whole body CT scan showed involvement of multiple retroperitoneal lymph nodes and bony metastases. On transrectal ultrasound examination, the prostate appeared homogeneous with no evidence of any local lesion. Biopsy showed him to have an adenocarcinoma of the prostate, Gleason score 3 + 4.

Treatment was started with the LHRH inhibitor goserelin acetate (Zoladex) at a dose of 10.8 mg subcutaneously every 12 weeks and the antiandrogen bicalutamide (Casodex) 50 mg daily per os. On this regimen, the patient experienced pain relief and his PSA fell to a nadir of 14 ng/mL.

By October 2006, his PSA had increased to 94 ng/mL and the bicalutamide was withdrawn. The patient continued to receive goserelin acetate without interruption. Two months following withdrawal of bicalutamide, the patient’s PSA rose to 180 ng/mL. Cyproterone acetate (Armocur) was started, but his PSA continued to rise to 245 ng/mL, with recurrence of his back pain. A bone scan performed in March 2007 showed increased uptake of 99m technetium in metastatic lesions in the ribs bilaterally, in thoracic vertebrae D9-D11, in the acetabulum, and in the left pubic ramus. A CT scan showed retroperitoneal lymphadenopathy and multiple sclerotic bone lesions.

Chemotherapy with vinorelbine was initiated, together with the bisphosphonate–zoledronic acid for his bone lesions. Goserelin acetate was continued. The patient demonstrated a good response to this regime, with disappearance of his pain, a fall in his PSA from 245 ng/mL at the start of treatment to 11 ng/mL, and disappearance of the retroperitoneal lymph nodes and bony metastases on CT scan. After 5 months, vinorelbine was withdrawn as the patient developed peripheral neuropathy with weakness of the proximal muscles of the legs. Following discontinuation of the drug, muscle strength gradually improved. The patient continued to receive goserelin acetate and zoledronic acid every 3 months, and he has remained asymptomatic on this drug combination, with PSA levels of around 11 ng/ mL, in the 33 months since chemotherapy was stopped.

## DISCUSSION

Most patients with advanced prostatic cancer initially show a good response to hormone therapy and this can persist for one to several years. Invariably, however, disease progression occurs and when this happens, unless additional measures are instituted, the life expectancy of the patient is only about a year. Why do patients become hormone resistant? Current theories suggest that during the course of the disease, new clones of cells are constantly emerging. The likelihood exists, therefore, that a clone that is nonhormone dependent will form and be uninfluenced by either LHRH agonists or antiandrogen drugs. This does not mean, of course, that there are not still many clones of cells that are hormone dependent and, for this reason, it is important to continue to administer hormone therapy to these patients. Recently, a *de novo* intratumoral androgen synthesis during progression of prostate cancer has been postulated[7,8]. This intratumoral *de novo* synthesis is presumed to maintain sufficient intracellular androgen levels for activating androgen receptor target genes. Thus, the tumor is enabled to survive in spite of low androgen serum levels. As a result, *de novo* androgen synthesis and androgen receptor overexpression after standard androgen deprivation therapy do not lead to a hormone refractory status, a term that is usually used for this situation.

Clearly, however, some additional intervention is necessary and the treatment of choice at this stage is a well-tolerated chemotherapeutic agent.

The standard chemotherapy for this purpose is currently docetaxel based, and two large randomized clinical trials have shown that treatment with this agent prolongs survival, improves symptoms, and reduces PSA[1,2]. There are also substantial data to suggest that “hormone-resistant” prostate cancer will continue to respond to hormone therapy even after the failure or withdrawal of docetaxel-based chemotherapy. Serrate et al. have demonstrated that diethylstilbestrol (DES) has activity in patients with progressive disease after first-line docetaxel. A PSA response as defined by a fall in PSA >30% and >50% was observed in five out of 20 patients (25%) and three out of 20 patients (15%), respectively[4].

Shamash et al. described the reinduction of hormone sensitivity in hormone refractory prostate cancer patients following chemotherapy with lomustine and chlorambucil. In their prospective study, 16 out of 43 patients (37%) who were rechallenged with hormone therapy following the failure of chemotherapy had a 50% PSA response to DES and dexamethasone. This response was associated with an improved overall survival (14.0 months vs. 3.7 months). Eight out of 12 patients who did not respond to DES before chemotherapy did so postchemotherapy. In this study, patients were not androgen suppressed during chemotherapy[5].

Cox and Sundar described two hormone-resistant patients who continued androgen-suppressive therapy throughout their treatment with docetaxel and prednisolone and who subsequently responded to rechallenge with DES[6].

In the patient described, we chose vinorelbine as first-line chemotherapy because of its good tolerability and because we wished to avoid provoking fluid retention in a patient with existing heart disease. We combined this with a bisphosphonate, since these compounds have been demonstrated to exert a direct inhibitory effect on both tumor growth and angiogenesis in preclinical studies[9]. We used zoledronic acid, which is a third-generation bisphosphonate that inhibits osteoclastic resorptive activity and has been shown to increase levels of apoptosis in prostate cancer cells[10]. In two recently reported clinical studies, ABCS[11] and Z/Zo-FAST[12], a disease-free survival benefit has been demonstrated in women receiving adjuvant endocrine therapy plus zoledronic acid. In patients with breast cancer who received neoadjuvant chemotherapy in combination with zoledronic acid, a reduction in the proportion of disseminated tumor cells detected in the bone marrow was noted at the time of surgery[13]. Analysis of a subset of the AZURE trial has shown that the addition of zoledronic acid to neoadjuvant chemotherapy improves tumor response, as judged by a more pronounced reduction in tumor size and an increased level of pathological complete response[14]. In our own patient, treatment with zoledronic acid may possibly account for part of the antitumor effect and clinical improvement observed.

Whereas much is understood regarding the ways in which androgen dependence may be lost, the mechanisms by which it can be regained are far less well defined. In particular, what is the precise role of chemotherapy in these circumstances? If it is postulated that both androgen-dependent and androgen-independent clones may exist side by side in a given tumor, the possibility exists that appropriate chemotherapy may selectively reduce the proportion of androgen-independent clones, while leaving the androgen-sensitive clones intact. This, in turn, would manifest itself clinically as an apparent restoration of hormone sensitivity. Other possible mechanisms cannot be ruled out and these include the possibility that certain chemotherapeutic agents may activate pathways that lead to a restoration of hormone sensitivity within the androgen receptor itself, or that they may induce changes in nonandrogen receptor activation influencing downstream survival pathways[15].

Our patient continued to receive goserelin during vinorelbine chemotherapy and responded with a dramatic fall in PSA, which remains low more than 2.5 years following withdrawal of the vinorelbine.

We suggest that chemotherapy with vinorelbine or docetaxel may attack both hormone-resistant and hormone-sensitive clones. When chemotherapy is withdrawn for any reason, the reinduction of sensitivity to hormone therapy may occur. Hormone therapy may thus be a viable option following treatment with a chemotherapeutic agent in patients with advanced prostatic cancer.
